# Inhibition of cancer growth *in vitro* and *in vivo* by a novel ROS-modulating agent with ability to eliminate stem-like cancer cells

**DOI:** 10.1038/cddis.2017.272

**Published:** 2017-06-22

**Authors:** Jiankang Wang, Bingling Luo, Xiaobing Li, Wenhua Lu, Jing Yang, Yumin Hu, Peng Huang, Shijun Wen

**Affiliations:** 1 School of Pharmaceutical Sciences, Sun Yat-sen University, Guangzhou, China; 2 Sun Yat-sen University Cancer Center; State Key Laboratory of Oncology in South China; Collaborative Innovation Center for Cancer Medicine, Sun Yat-sen University, Guangzhou, China

## Abstract

Reactive oxygen species (ROS) have a crucial role in cell signaling and cellular functions. Mounting evidences suggest that abnormal increase of ROS is often observed in cancer cells and that this biochemical feature can be exploited for selective killing of the malignant cells. A naturally occurring compound phenethyl isothiocyanate (PEITC) has been shown to have promising anticancer activity by modulating intracellular ROS. Here we report a novel synthetic analog of PEITC with superior *in vitro* and *in vivo* antitumor effects. Mechanistic study showed that LBL21 induced a rapid depletion of intracellular glutathione (GSH), leading to abnormal ROS accumulation and mitochondrial dysfunction, evident by a decrease in mitochondrial respiration and transmembrane potential. Importantly, LBL21 exhibited the ability to abrogate stem cell-like cancer side population (SP) cells in non-small cell lung cancer A549 cells associated with a downregulation of stem cell markers including OCT4, ABCG2, SOX2 and CD133. Functionally, LBL21 inhibited the ability of cancer cells to form colonies *in vitro* and develop tumor *in vivo*. The therapeutic efficacy of LBL21 was further demonstrated in mice bearing A549 lung cancer xenografts. Our study suggests that the novel ROS-modulating agent LBL21 has promising anticancer activity with an advantage of elimination of stem-like cancer cells. This compound merits further study to evaluate its potential for use in cancer treatment.

Cancer cells exhibit various differences in cellular biological activities, including the level of reactive oxygen species (ROS), which is involved in redox balance, cellular proliferation, cancer progression and cancer stem cells (CSCs).^1, 2^ The elevated level of ROS in cancer cells is often considered as an adverse factor to cause genetic instability. Mitochondria act as the major source of cellular ROS, and excessive generation of ROS in turn causes mitochondrial dysfunction.^3, 4^ Meanwhile, intracellular ROS accumulation induces cell cycle arresting at G1 or G2/M phase and thereby impedes cell proliferation.^5, 6^ Mounting evidences demonstrate that malignant cancer cells show abnormal increase of ROS level with high oxidative stress, which in turn expose these cancer cells more vulnerable to further oxidative stress. Therefore, targeting ROS may provide a novel cancer therapeutic strategy to kill malignant tumor cells and hold a great promise.^5^

In recent years, growing evidences suggest that CSCs, a small subgroup of special cancer cells, have high self-renewal capability and have important roles in maintaining tumor growth and causing drug resistance.^7, 8^ However, CSCs in tumors contain low ROS levels compared with non-tumorigenic cells because of their increased antioxidant capacity to defend ROS stress.^9^ As a consequence, pharmacologically targeting ROS defense system, for example, excessively increasing ROS level could reduce clonogenicity and mediate death of CSCs.^10^

Phenethyl isothiocyanates (PEITC), a naturally occurring compound widely present in cruciferous vegetables, is able to modulate excessive ROS generation and selectively kill cancer cells.^11^ Previous studies have demonstrated that PEITC killed malignant cancer cells by disabling glutathione (GSH) antioxidant system and disrupting redox-sensitive survival pathways.^10, 12^ Moreover, PEITC has been registered in clinical trials for cancer prevention and treatment (ClinicalTrials.gov Identifiers: NCT00005883, NCT00691132 and NCT01790204), indicating that PEITC could hold a therapeutic promise to treat cancer patients. In our recent work, using PEITC as the lead compound, a series of small molecular analogs of PEITC were designed and synthesized in order to discover more potent ROS modulator with better anticancer capacity (data not shown). Finally, *N*-ethyl-4-(2-isothiocyanatoethyl)benzamide (labeled as LBL21) was found to show a best anti-proliferative ability. The following *in vitro* studies demonstrated that LBL21 caused more ROS accumulation, mitochondrial dysfunction and CSCs elimination. The anticancer effect is further demonstrated in subsequent *invivo* study with mice bearing A549 lung cancer xenografts.

## Results

### Structure modification of PEITC led to discovery of LBl21 with better anticancer potency

**Figure 1:**
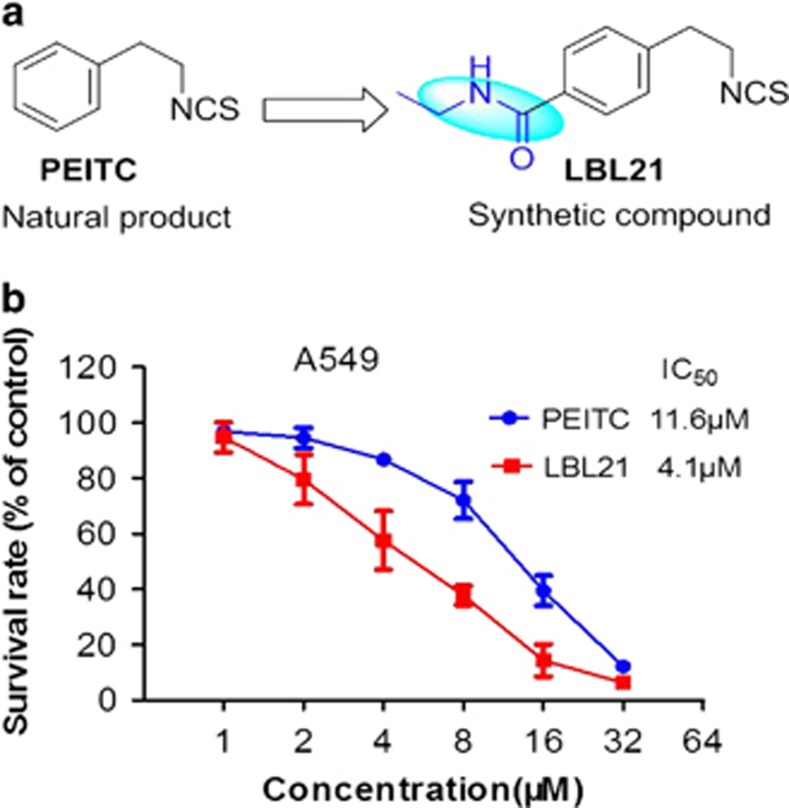
LBL21 and its anti-proliferation effect on lung cancer cells A549. (**a**) Structures of PEITC and LBL21; (**b**) LBL21 and PEITC inhibited the proliferation of A549 at dose dependence. Bars means±S.D. *n*=3

PEITC is one of natural occurring isothiocyanates, which are enriched in cruciferous vegetables and have showed cancer prevention and therapeutics effects.^13, 14, 15^ On one hand, PEITC selectively killed malignant cancer cells via ROS modulation mechanism.^10^ On the other hand, few of PEITC and its analogs have showed IC_50_ value against various cancer cells at low micromolar concentration. Thus, it is of importance to discover more potent isothiocyanates.^16, 17^ Using PEITC as the lead compound, a series of small molecule analogs of PEITC were designed and synthesized (data not shown). Compared with PEITC, LBL21 has an ethylamide group at the *para* position of benzene ring of PEITC (Figure 1a). However, the addition of this special functional group substantially increased the potency of LBL21 to kill lung cancer cell line A549, with IC_50_ value from 11.6 *μ*M for PEITC down to 4.1 *μ*M for LBL21 (Figure 1b).

### LBL21 caused more cytotoxicity in several cancer cell lines

**Figure 2:**
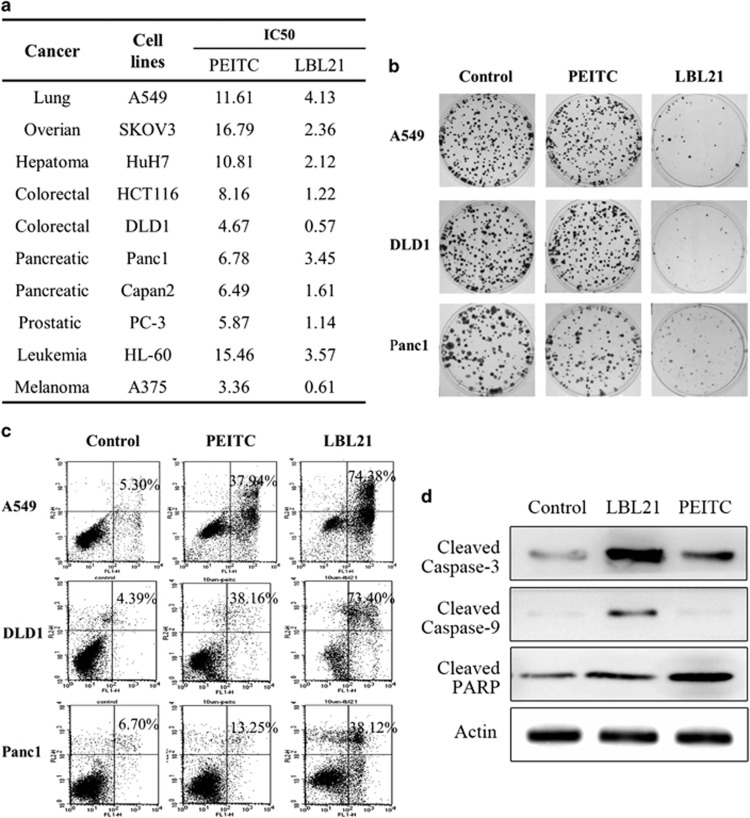
Cytotoxicity of LBL21 in various human cancer cell lines. (**a**) MTS assay was performed to evaluate the effect of PEITC and LBL21 on the proliferation of different cancer cell lines after 72-h treatment. The errors in determinations of IC_50_ are within ±10% of their value. (**b**) The image of colony formation of A549, DLD1 and Panc1 after 14 days treatment with PEITC or LBL21 at 1 *μ*M. (**c**) Apoptosis rates were determined by annexin-V/PI assay after cancer cells were treated with PEITC or LBL21 at 10 *μ*M for 72 h. Percentage numbers indicate annexin-V/PI-positive cell fraction. (**d**) The protein expression levels of the apoptotic markers after 72- h treatment in A549 cells

To validate *in vitro* anticancer effect of LBL21 could be broad, we first tested its cellular growth inhibition on the other cancer cells using MTS assay (Figure 2a). Among different types of cancer cell lines including lung cancer (A549), colorectal cancer (DLD1) and pancreatic cancer (Panc1 and Capan2), LBL21 showed potent anti-proliferation at submicromolar concentrations after 72-h treatment. More importantly, LBL21 consistently exhibited much lower IC_50_ values in all the tested cancer cell lines, with 3–10 times of anticancer potency compared with PEITC. These results implied that our previous structure modification indeed significantly increased anticancer capability of PEITC.

Then colony formation assay was then performed to further investigate anti-proliferation effect of LBL21 (Figure 2b). Three cancer cell lines A549, DLD1 and Panc1 were incubated with either PEITC or LBL21 at the same concentration (1 *μ*M) for 14 days. LBL21 almost completely restrained the colony formation of all three cancer cell lines whereas there was little impact by PEITC treatment, implying that LBL21 induced more inhibitory effect of long-time cellular proliferation. In the following apoptosis measurement, DLD1, Panc1 and A549 cancer cells were treated with PEITC or LBL21 at 10 *μ*M for 72 h, and then apoptotic rates were determined by flow cytometer (Figure 2c). PEITC only caused 15–40% cell fatality, whereas LBL21 induced 40–80% apoptotic rate, 2–3 times potency of PEITC. The cleavage of caspase-3, caspase-9 and PARP further demonstrated that LBL21 could induce cell apoptosis (Figure 2d).

### LBL21 substantially caused redox imbalance

**Figure 3:**
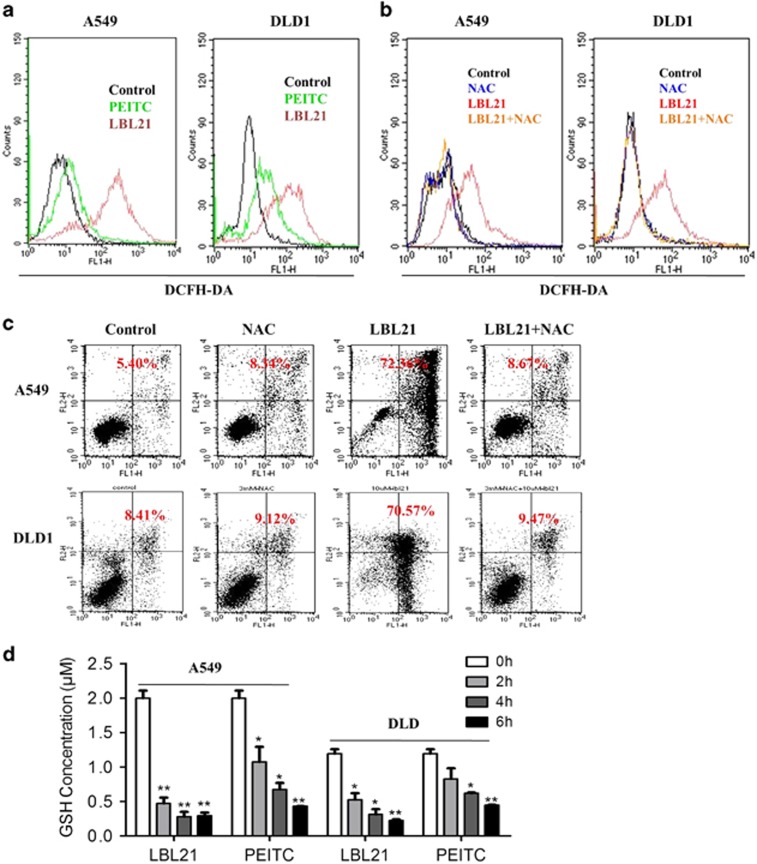
LBL21 caused ROS increase and GSH depletion. (**a**) DCF-DA staining showed the effect of PEITC (green line) and LBL21 (red line) at 10 *μ*M on cellular ROS levels after 6 h treatment. (**b**and **c**) Pretreatment with NAC at 3 mM effectively reversed the ROS accumulation (6 h) and apoptosis rates (48 h) induced by LBL21 at 10 *μ*M. Percentage numbers indicate cell death population. (**d**) Cellular GSH contents after 10 *μ*M LBL21 or PEITC treatment for indicated hours. Bars means ±S.D. *P<0.05, **P<0.01 *versus* control, *n*=3

As LBL21 is an analog of natural product PEITC, it may cause cell death by a similar ROS-modulating mechanism, which is well established for PEITC. First, we tested ROS level alteration in lung and colorectal cancer cell lines after drug treatments using DCF-DA staining (Figure 3a). After 6-h treatment, 10 *μ*M LBL21 induced tremendous accumulation of ROS relative to PEITC, consistent with our cytotoxicity results. It is well known that excessive ROS production can cause severe cytotoxicity depending on the ROS level and the defense of ROS stress by intracellular antioxidants.^1^ To investigate whether the increased ROS was the primary cause of cell death, *N*-acetyl-l-cysteine (NAC), a ROS scavenger was used to act as antioxidant agent. DCF-DA fluorescence revealed that pretreatment of 3 mM NAC significantly decreased the ROS accumulation caused by LBL21 in A549 and DLD1 cells (Figure 3b). Importantly, pretreatment of NAC totally reversed the cell apoptosis induced by LBL21, as shown in Figure 3c. It is worth noting that NAC itself did not interfere with ROS or apoptosis levels. The results implied that NAC repaired cellular redox imbalance, which was primarily caused by LBL21.

GSH that can maintain intracellular redox state by scavenging ROS, is considered as a key cellular endogenous molecule in the antioxidant pathways.^18^ As LBL21 significantly impacted the cellular redox balance, it may intervene with GSH level. As shown in Figure 3d, LBL21 drastically decreased cellular GSH level in a time-dependent manner, and GSH depletion was observed at early time after 2-h treatment. Importantly, LBL21 exhibited stronger GSH depletion effect compared with PEITC, consistent to the result that LBL21 induced more increase of ROS.

### LBL21 disturbed mitochondrial function

**Figure 4:**
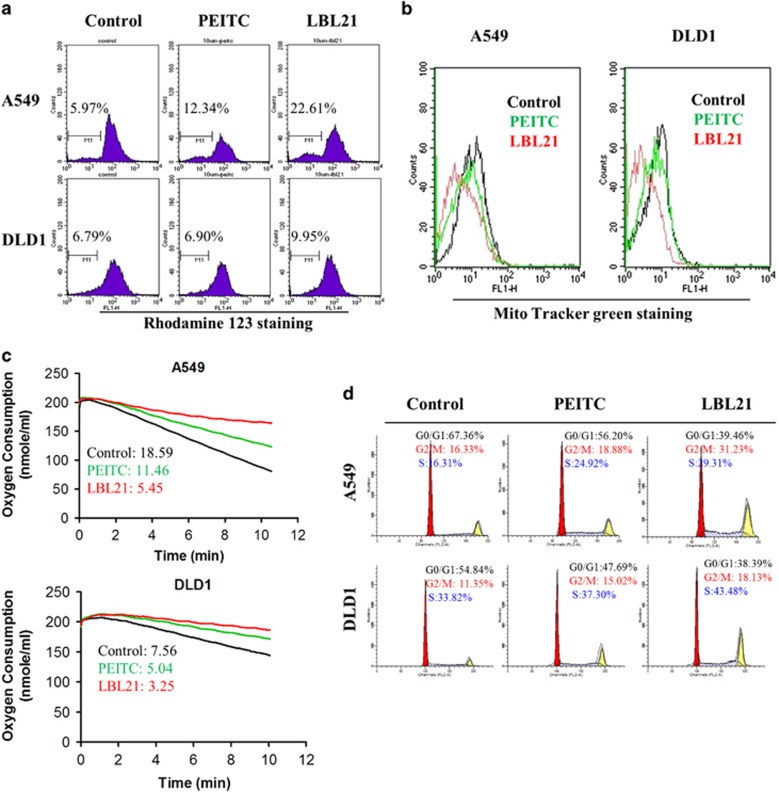
LBL21 regulated mitochondrial function and cell cycle. (**a**) The change of MTP after treatments of indicated drugs at 10 *μ*M, analyzed by flow cytometry using fluorescent dye Rhodamine-123. Percentage numbers showing Rhodamine-123-negative cell fraction. (**b**) The change of mitochondrial mass after treatments of indicated drugs at 10 *μ*M, determined by Mito Tracker Green staining. (**c**) The oxygen consumption rate after treatments of indicated drugs at 10 *μ*M for 6 h at 37 °C, measured using a Clark-type oxygen electrode. Numbers indicate quantitative O_2_ consumption rates. (**d**) Effect of PEITC and LBL21 at 10 *μ*M on the cell cycle of cancer cell lines for 24 h. Cellular DNA contents were measured by flow cytometry. The numbers indicate the normalization of each phase of cell cycle

The loss of mitochondrial transmembrane potential (MTP) is considered as a marker of mitochondrial dysfunction and an early signal of mitochondrial-initiated cell death.^19^ Rhodamine-123, a cell-permeable dye, can be sequestered by active mitochondria in proportion to MTP, and it is commonly used for monitoring membrane potential.^19^ As excessive ROS can influence mitochondrial functions, our new synthetic ROS-modulating agent, LBL21 may impact MTP. The Rhodamine-123 staining experiments revealed that LBL21 treatment group showed more percentage of lower fluorescence intensity especially in A549 cancer cells (Figure 4a), indicating that LBL21 could cause a loss of cellular MTP. The observed loss of MTP induced by LBL21 was much more than by PEITC. It is well established that mitochondria are the major intracellular source and primary target of ROS, so LBL21-mediated ROS increase may cause mitochondrial dysfunction. MitoTracker Green, an important tool for determining mitochondrial mass, can accumulate in mitochondrial regardless of the membrane potential.^20^ Using MitoTracker Green staining, we observed a significant decrease of mitochondrial mass with LBL21 treatment (Figure 4b), suggesting that the oxidative stress caused by LBL21 could lead to mitochondria mass decrease in cancer cells. Meanwhile, cellular oxygen consumption was also determined to validate our observed metabolic dysfunction caused by LBL21 (Figure 4c). Treatment of cancer cells with 10 *μ*M LBL21 (red line) caused mitochondrial respiration inhibition, as evidenced by the significant decrease of oxygen consumption in cancer cells. Exposure to LBL21 resulted in an approximately 60–70% reduction of the oxygen consumption rate, whereas PEITC at the same concentration caused only 30% reduction.

It has been reported that ROS could compromise the cell cycle regulatory function and then contribute to the uncontrolled cell proliferation.^6^ Thus, the effect of LBL21 and PEITC on the cell cycle progression were determined by flow cytometer. As shown in Figure 4d, LBL21 treatment led to G2/M arrest at 24 h significantly, compared with PEITC group.

### LBL21 effectively suppressed CSCs *in vitro* and tumor formation *in vivo*

**Figure 5:**
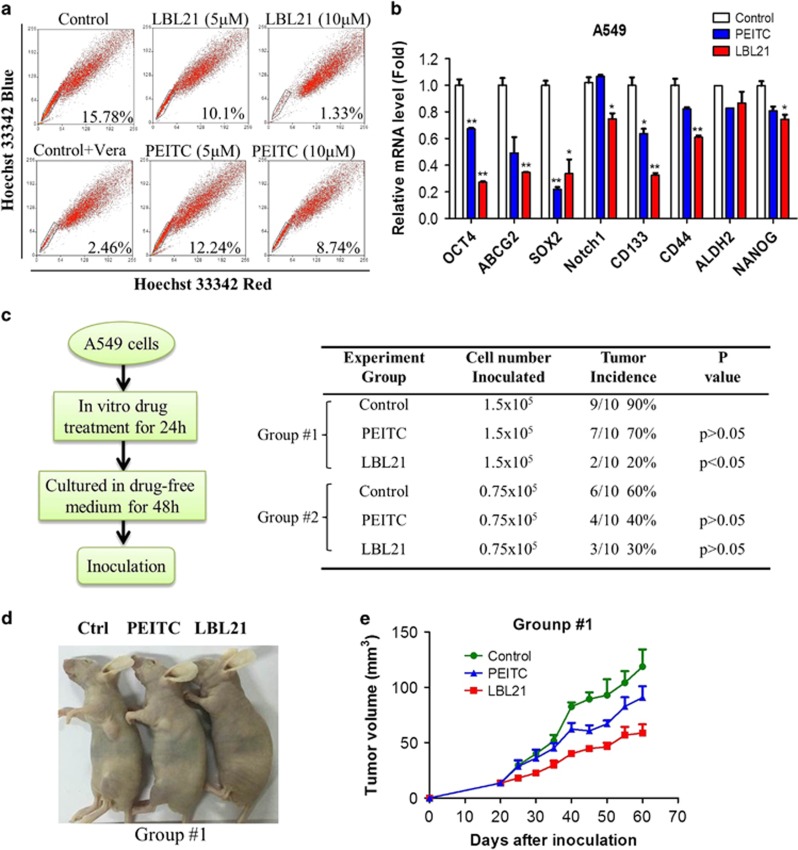
LBL21 suppressed CSCs *in vitro* and tumor formation *in vivo*. (**a**) A549 cells were incubated with LBL21 or PEITC for 24 h at indicated concentrations, and then SP cells were quantified by flow cytometry analysis. SP cells appeared as a dim tail with low red and blue Hoechst signals because of active efflux of the dye Hoechst 33342 (framed). Inhibition of the efflux pump by verapamil (Vera) led to cellular accumulation of Hoechst 33342, and SP sub-population disappeared. (**b**) mRNA expression levels of CSC biomarkers. A549 cells with different treatments for 24 h were analyzed via real-time qPCR. Bars means±S.D. *P<0.05, **P<0.01 *versus* control, *n*=3. (**c**) A549 cells were pretreated with 10 *μ*M PEITC or LBL21 for 24 h. Then the cells were washed and cultured in fresh medium for another 48 h to allow cell death occur. Equal viable cells were inoculated subcutaneously into the flanks of athymic mice using 1.5 × 10^5^ or 0.75 × 10^5^ cells per injection site. The mice were monitored for tumor incidence. (**d**) Images of tumors from representative mice in each group. (**e**) Measurement of tumor volume was carried out on the indicated days. Bars means±S.D.

Targeting CSCs is a promising strategy to eradicate cancers and overcome drug resistant.^8^ Side population (SP) cells within tumors are a subset of cancer cells with stem-like properties that can be identified by flow cytometry analysis because of their highly expressed ABC transporters.^21^ It is reported that having low ROS levels is crucial to maintain the sub-population of CSCs in cancers.^22^ Thus, we envisioned that our synthetic compound LBL21 would effectively eliminate SP cells via modulating ROS accumulation. SP cells were detected based on their ability to exclude Hoechst 33342 dye. These cells appeared as a distinct dim ‘tail’ in the flow cytometry plots, which revealed that the percentage of SP cells.^21^ Subsequent flow cytometry analysis showed that the incubation of A549 cells with LBL21 had a significant impact on the survival of SP cells (Figure 5a). In all, 5 *μ*M LBL21 obviously reduced the proportion of SP cells (from 15.8 to 10.1%) and 10 *μ*M almost completely killed SP cells (from 15.8 to 1.3%). Again, LBL21 exhibited stronger effect on the killing of SP cells than PEITC, which was consistent with its capacity of GSH depletion and ROS upregulation. Moreover, the qPCR analysis indicated that LBL21 caused a significant decrease in various CSC biomarkers CD44, CD133, OCT4, ABCG2, SOX2, ALDH2 and NANOG in mRNA expression levels (Figure 5b). Our results suggested that LBL21 may be used as a drug candidate to eradicate CSCs.

Based on the evidence that LBL21 could eliminate SP cells, we next studied the *in vivo* tumorigenicity of LBL21 pretreated A549 cells. As shown in Figure 5c, A549 cells were incubated with LBL21 or PEITC for 24 h, and then cultured in drug-free medium for another 48 h to allow time for the occurrence of cell death as well as the recovery of viable cells. After removing the detached dead cells, equal numbers of viable cells from various treatments were subcutaneously inoculated into the flanks of athymic mice. It is worth mentioning that A549 pretreated with the agents at same concentrations for 24 h did not cause obvious apoptosis (Supplementary Figure 1). The tumor formations were monitored continuously without further drug treatments. With the inoculated 1.5 × 10^5^ viable cells per injection site (group #1), 90% of the mice in blank control group and 70% of the mice in the PEITC treatment group developed tumors. However, LBL21 treatment substantially reduced the tumor incidence to 20%. In the other set of study in which the mice were inoculated with 0.75 × 10^5^ viable cells (group #2), 60% mice in the blank control group developed tumors, whereas the incidence of tumor formation was only 30% in LBL21 treated mice. Meanwhile, the growth of the developed tumors was also substantially prevented by LBL21 pretreatment (Figures 5d and 5e). The significant decrease of tumor formation and growth in the pretreated cancer cells may result from LBL21-mediated elimination of SP cells.

### LBL21 substantially suppressed tumor growth in A549 xenograft mice

**Figure 6:**
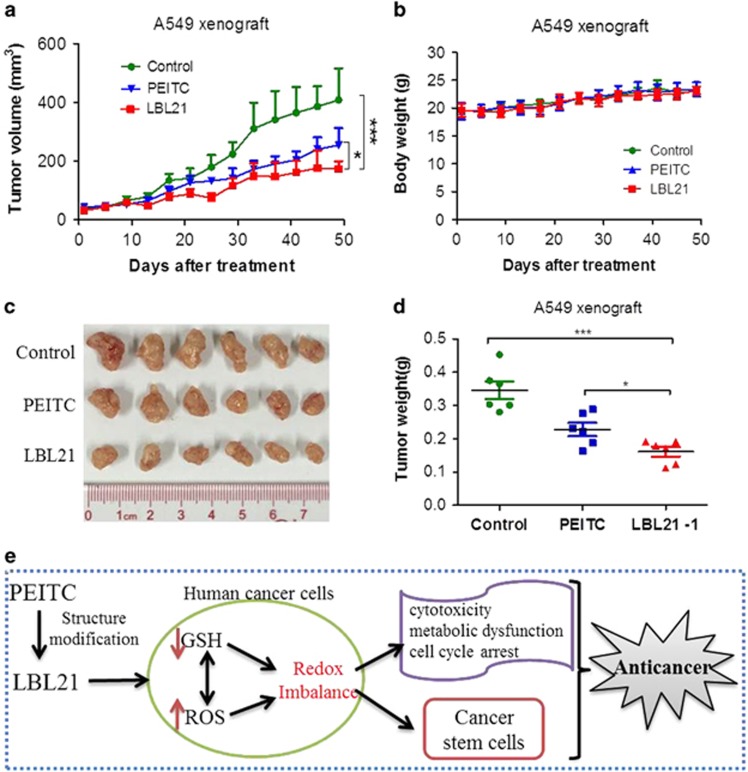
LBL21 inhibited tumor growth on A549 xenograft study. Immune-deficient BALB/c nude mice were utilized to establish A549 xenograft. Both PEITC and LBL21 were administered three times per week intraperitoneally at 25 mg/kg dosage. (**a** and **b**) The tumor volume and mice weight were determined twice a week. (**c**and**d**) Tumor weight was measured after the mice were killed. Bars means±S.D. *P<0.05, ***P<0.001, *n*=6. (**e**) A proposed mechanism model for our molecule LBL21

As LBL21 pretreatment prevented the tumorigenicity of A549 cancer cells, we next investigated whether LBL21 could therapeutically inhibit the tumor growth in A549 xenograft mice. Thus, nude mice were subcutaneously injected in the flanks with A549 cells and randomly assigned to three groups (six mice per group). Compared with the blank control, the mice treated with LBL21 three times weekly at 25 mg/kg dosage had tumor growth substantially reduced, with >50% volume inhibition (Figure 6a). Meanwhile, no significant weight loss or illness was observed in any tested mouse during the treatments (Figure 6b), indicating LBL21 at the designed dose was safe. Finally, mice were killed to obtain their tumors after 50 days treatment. As shown in Figures 6c and 6d, LBL21 treatment markedly decreased >60% tumor weight. PEITC treatment showed moderate effect on inhibiting the tumor growth and tumor weight in the tested mice.^23^ Overall, our new synthetic compound LBL21 exhibited significant *in vivo* anti-proliferative activity.

## Discussion

ROS are considered as one of the most important chemically reactive molecules in regulation of cell functions. Moderate increase of ROS can promote cell differentiation and proliferation, whereas excessive ROS accumulation will cause oxidative stress and damage to cells.^5^ It is well known that malignant cancer cells have increased ROS level and are more dependent on endogenous antioxidant to maintain a redox balance for cell survival, suggesting that pharmacological ROS modulation could be exploited to gain targeted therapeutic benefits.^24^ Naturally occurring compound PEITC and other isothiocyanate compounds can conjugate with endogenous GSH, causing a depletion of GSH and subsequent oxidative stress and cytotoxicity.^10^ Some of these natural iosthiocyanates are under clinical trials for cancer prevention and cancer therapy. To further improve anticancer potency of PEITC, we designed and synthesized a series of PEITC analogs to seek a better drug candidate. One of PEITC analogs, LBL21 has exhibited strongest anticancer effect in various cancer cell lines including lung cancer, colorectal cancer and pancreatic cancer.

In the following study, LBL21 substantially depleted intracellular antioxidant GSH and modulated ROS increase, which may be a primary factor to cause cancer cell death.^5, 25, 26^ A ROS scavenger NAC was able to reverse the cell apoptosis caused by LBL21, further suggesting LBL21-induced cell death may result from excessive ROS production. Abnormal oxidative stress can cause damage to mitochondrial function.^27^ We observed that LBL21 induced MTP loss, decreased mitochondrial mass and suppressed oxygen consumption of A549 cancer cells. MTP is an important parameter of mitochondrial function^19^ whereas mitochondrial mass also reflect mitochondrial proliferation and function.^20^ Thus, the mitochondrial dysfunction caused by LBL21 further inhibited aerobic respiration, thereby leading to more cell apoptosis. Meanwhile, cell cycle arrest and apoptosis are closely regarded as cellular protection mechanisms under oxidative stress.^6^ Some previous studies have revealed that PEITC and other natural isothiocyanates could regulate several cyclins and cause G2/M phase arrest,^12, 28, 29^ which is associated with increased apoptosis.^30^ Our current study demonstrated that LBL21 caused more cell arrest at G2/M relative to PEITC.

CSCs are critical in tumor initiation, development, metastasis and recurrence. Previous studies demonstrate that increasing ROS may be potentially effective to kill CSCs, whereas CSCs highly depend on antioxidant system to maintain a low ROS level.^9^ Lung cancer is the most frequent cause of cancer patient death around the world, especially in China.^31^ A549, a non-small lung cancer cell line contains a small sub-population of special cells with stem-like properties, called SP cells, with self-renewal capability.^32, 33^ Our study showed that LBL21 substantially reduced SP cells in A549, and downregulated a series of genes, which are regarded as CSC biomarkers. Pretreatment of LBL21 greatly impaired tumorigenicity of A549 cells in nude mice. Further *in vivo* study showed that LBL21 markedly inhibited the tumor growth without side effects. At the same drug weight doses, LBL21 showed stronger *in vivo* cancer therapeutic effect than PEITC. However, it is worth noting that molecular weight of LBL21 (234) was higher than that of PEITC (163), implying that equally weighed LBL21 actually provided less molar quantity but with stronger tumor suppression.

In summary, we report a novel synthetic PEITC analog LBL21 that killed malignant cancer cells by intervening cellular redox balance via ROS modulation (Figure 6e). LBL21 exhibited significant anti-proliferation effect both *in vitro* and *in vivo*, and such effects were much better than PEITC. It also substantially reduced CSC-like SP cells and CSC biomarkers in lung cancer cell line A549. As several clinical trials with PEITC have been registered with the US National Institutes of Health, our new discovered compound LBL21 may hold a therapeutic promise to treat malignant tumors.

## Materials and methods

### Cell lines and cell culture

All the cells used in this research were obtained from American Type Culture Collection (ATCC, Rockville, MD, USA) and were cultured under the conditions specified by the manufacturer. All media were obtained from Invitrogen Life Technologies (Carlsbad, CA, USA) and supplemented with 10% fetal bovine serum (GIBCO, Carlsbad, CA, USA).

### Reagents

PEITC, NAC, CM-H_2_DCF-DA, Hoechst 33342, verapamil, Rhodamine-123 and Mito Tracker green were obtained from Sigma-Aldrich (St. Louis, MO, USA). LBL21 were synthesized in our laboratory.

### Cell growth and apoptosis measurement

Cell proliferation was measured by MTS assay (Promega, Madison, WI, USA). Cancer cells were incubated in 96-well plate with indicated agents for 72 h, followed by 4- h incubation with 20 *μ*l MTS solution. The spectrophotometric absorbance was determined by microplate reader (Thermo, Helsinki, Finland) at 490 nm. Apoptotic rates were determined by an Annexin-V/PI assay kit (KeyGEN, Nanjing, China). After the treatment with various agents, cells were collected and stained with Annexin-V-FITC and propidium iodide (PI) and apoptotic rates were measured using FACS flow cytometer (BD Biosciences, San Diego, CA, USA).

### Colony formation

Cancer cells were seeded in a six-well plate (500 cells per well) and incubated with indicated agents for 14 days. The colonies were stained with crystal violet after fixation. Then, the number of colonies was counted by AlphaImager HP system (ProteinSimple, Santa Clara, CA, USA).

### The measurement of ROS levels, MTP and mitochondrial mass

After 24-h treatment of various agents, cells were collected and stained with different dyes, followed by flow cytometry analysis using FACS Calibur flow cytometer (BD Biosciences). Cellular ROS levels were stained by DC-FHDA (Sigma-Aldrich), MTP stained by Rhodamine-123 (Sigma-Aldrich) and mitochondrial mass stained by Mito Tracker green (Sigma-Aldrich).

### GSH measurement

GSH-Glo Glutathione Assay kit (Promega) was utilized to measure cellular GSH levels. After treatment with various agents, the medium was removed from the wells. Then cells were incubated with 100 *μ*l mixed GSH-Glo reagent for 30 min and then 100 *μ*l Luciferin Detection Reagent for another 15 min. Luminescent signals were detected using a Fluoroskan luminescence scanner (Thermo Fisher, Rockford, IL, USA).

### Cell cycle

Cells in logarithmic phase were seeded in six-well plates and incubated with different agents for 24 h. Then cells were collected, fixed with 75% alcohol at 4 °C for 4 h and incubated with RNase at 37 °C for 30 min followed by addition of PI. Cell cycle distribution was measured using FACS Calibur flow cytometer (BD Biosciences).

### Cellular oxygen consumption assay

Cells in equal number were suspended in fresh medium at 37 °C pre-equilibrated with 21% oxygen and transferred to a sealed respiration chamber equipped with a Clark oxygen sensor, a thermostat and a micro-stirring device (Oxytherm, Hansatech Instrument, Norfolk, UK). Detection was performed with constant stirring at 37 °C. Oxygen consumption rate was in nanomoles of O_2_ consumed per min per 5 million cells.

### Detection of SP cells and non-SP cells

Hoechst 33342 staining and flow cytometer were utilized to detect SP and non-SP cells. Briefly, cells were collected and re-suspended in pre-warmed RPMI 1640 medium containing 2% FBS at a density of 1 × 10^6^ cells/ml. Then cells were stained using following modification, which was reported by previous study:^21, 34^ cancer cells were incubated with Hoechst 33342 (5 *μ*g/ml) with or without the ABC transporter inhibitor verapamil for 90 min at 37 °C with intermittent shaking in the darkness. The cells were washed with cold PBS and kept at 4 °C, followed by flow cytometry analysis, which was performed using MoFlo XDP Cell Sorter (Beckman Coulter, Brea, CA, USA).

### Quantitative real-time PCR

After various treatments for 24 h, cells were collected to obtain total RNA using Trizol reagent (Invitrogen, Carlsbad, CA, USA). The reverse transcription was done following the manufacturer’s protocol of PrimerScript RT reagent kit (TaKaRa, Dalian, China). Then, quantitative real-time PCR was performed using a real-time PCR measurement system CFX96 (Bio-Rad, Richmond, CA, USA) based on SYBR Premix Ex TaqII. All samples were analyzed at least twice. The specific primers were shown in Supplementary Table S1.

### Evaluation of *in vivo* tumorigenicity

A549 cells were pretreated with indicated agents at 10 *μ*M for 24 h, washed and cultured in drug-free fresh medium for another 48 h. The viable cells were collected and injected subcutaneously into the flanks of 5-week-old female immune-deficient BALB/c nude mice (Vital River, Beijing, China) at 1.5 × 10^5^ cells or 0.75 × 10^6^ cells per injection site. The presence or absence of a visible or palpable tumor was evaluated and tumor growth was monitored twice a week. Tumor volume was calculated by the formula 0.5 × length × width^2^. This animal study and the followed were performed in compliance with a research protocol approved by the Ethics Committee of Sun Yat-sen University Cancer Center.

### A549 xenograft study

In all, 2 × 10^6^ exponentially growing A549 cells re-suspended in 0.1 ml PBS were subcutaneously injected to 5-week-old female immune-deficient BALB/c nude mice (Vital River). The drug treatment was started when the mice formed palpable tumors. Both LBL21 and PEITC were formulated in 80% PBS, 10% DMSO and 10% cremophor. These two agents were administered intraperitoneally three times per week at a dose of 25 mg/kg. The mice body weight and tumor volume, which was calculated as 0.5 × length × width^2^ were measured twice a week. At the end of the experiment, mice were killed and their tumor weights were measured after resection.

### Statistical analysis

All these data were presented as means±S.D. Comparison of difference between two groups was evaluated by Student’s *t-*test. The difference between more than two groups was determined by one-way ANOVA (Prism GraphPad, San Diego, CA, USA). *P*< 0.05 was considered statistically significant.
