# Supplementary material for: Inhibition of cancer growth in vitro and in vivo by a novel ROS-modulating agent with ability to eliminate stem-like cancer cells
Source: Cell Death Dis. 2017 Jun 22;8(6):e2887–. doi: 10.1038/cddis.2017.272 (PMC5520927; doi:10.1038/cddis.2017.272)
Supplement: Supplementary Material [file cddis2017272x1.doc]

**Supplemental Table S1. The sequences of primers used for SYBR Green real-time PCR**

| **Gene** | **Forward** | **Reverse** |
| --- | --- | --- |
| OCT4 | CTGGGTTGATCCTCGGACCT | CCATCGGAGTTGCTCTCCA |
| ABCG2 | CAGGTGGAGGCAAATCTTCGT | ACCCTGTTAATCCGTTCGTTTT |
| SOX2 | GCCGAGTGGAAACTTTTGTCG | GGCAGCGTGTACTTATCCTTCT |
| Notch1 | GAGGCGTGGCAGACTATGC | CTTGTACTCCGTCAGCGTGA |
| CD133 | AGTCGGAAACTGGCAGATAGC | GGTAGTGTTGTACTGGGCCAAT |
| CD44 | CTGCCGCTTTGCAGGTGTA | CATTGTGGGCAAGGTGCTATT |
| ALDH2 | ATGGCAAGCCCTATGTCATCT | CCGTGGTACTTATCAGCCCA |
| NANOG | TTTGTGGGCCTGAAGAAAACT | AGGGCTGTCCTGAATAAGCAG |
| Beta-actin | TTCTACAATGAGCTGCGTGTG | GGGGTGTTGAAGGTCTCAAA |

**Supplementary Figure 1.** Apoptosis rates were determined by annexin-V/PI assay after cancer cells were treated with PEITC or LBL21 at 10 μM for 24h. Percentage numbers indicate annexin-V/PI positive cell fraction.
